# Early presentation of gait impairment in Wolfram Syndrome

**DOI:** 10.1186/1750-1172-7-92

**Published:** 2012-12-08

**Authors:** Kristen A Pickett, Ryan P Duncan, James Hoekel, Bess Marshall, Tamara Hershey, Gammon M Earhart

**Affiliations:** 1 Program in Physical Therapy, Washington University in St. Louis, Campus Box 8502, 4444 Forest Park Blvd, St. Louis MO 63108, MO, USA; 2 Department of Neurology – Movement Disorders Section, Washington University School of Medicine, St Louis, MO, USA; 3 Department of Ophthalmology, Washington University School of Medicine, St. Louis, MO, USA; 4 Department of Pediatrics, Washington University School of Medicine, St Louis, MO, USA; 5 Department of Psychiatry, Washington University School of Medicine, St Louis, MO, USA; 6 Department of Cell Biology, Washington University School of Medicine, St Louis, MO, USA; 7 Department of Radiology, Washington University School of Medicine, St. Louis, MO, USA; 8 Department of Anatomy & Neurobiology, Washington University School of Medicine, St. Louis, MO, USA

**Keywords:** Wolfram Syndrome, Gait, Ataxia, Development

## Abstract

**Background:**

Classically characterized by early onset insulin-dependent diabetes mellitus, optic atrophy, deafness, diabetes insipidus, and neurological abnormalities, Wolfram syndrome (WFS) is also associated with atypical brainstem and cerebellar findings in the first decade of life. As such, we hypothesized that gait differences between individuals with WFS and typically developing (TD) individuals may be detectable across the course of the disease.

**Methods:**

Gait was assessed for 13 individuals with WFS (min 6.4 yrs, max 25.8 yrs) and 29 age-matched, typically developing individuals (min 5.6 yrs, max 28.5 yrs) using a GAITRite ® walkway system. Velocity, cadence, step length, base of support and double support time were compared between groups.

**Results:**

Across all tasks, individuals with WFS walked slower (p = 0.03), took shorter (p ≤ 0.001) and wider (p ≤ 0.001) steps and spent a greater proportion of the gait cycle in double support (p = 0.03) compared to TD individuals. Cadence did not differ between groups (p = 0.62). Across all tasks, age was significantly correlated with cadence and double support time in the TD group but only double support time was correlated with age in the WFS group and only during preferred pace forward (r_s_= 0.564, p = 0.045) and dual task forward walking (r_s_= 0.720, p = 0.006) tasks. Individuals with WFS also had a greater number of missteps during tandem walking (p ≤ 0.001). Within the WFS group, spatiotemporal measures of gait did not correlate with measures of visual acuity. Balance measures negatively correlated with normalized gait velocity during fast forward walking (rs = −0.59, p = 0.03) and percent of gait cycle in double support during backward walking (rs = −0.64, p = 0.03).

**Conclusions:**

Quantifiable gait impairments can be detected in individuals with WFS earlier than previous clinical observations suggested. These impairments are not fully accounted for by the visual or balance deficits associated with WFS, and may be a reflection of early cerebellar and/or brainstem abnormalities. Effective patient-centered treatment paradigms could benefit from a more complete understanding of the progression of motor and other neurological symptom presentation in individuals with WFS.

## Background

Diabetes insipidus, diabetes mellitus, and optic atrophy (DIDMOAD) or Wolfram syndrome (WFS) is associated with a host of symptoms [1-4] including diabetic, psychiatric [5], neurologic, opthalmologic [6] and urologic [7] complications. An autosomal recessive disease with a reported prevalence between 1 in 100,000 [8] and 1 in 700,000 [2], the diagnosis of WFS is confirmed via presence of one or both of the identified WFS genes [9-12]. Complications of the disorder result in reduced quality of life and premature death, with a reported median life expectancy of 30 years [2]. Currently, effective interventions are lacking and there is no known cure for WFS.

The brainstem and cerebellum are thought to be particularly vulnerable to the neurodegenerative process in WFS [2]. In a single case of WFS, Pakdemirli et al. [13] reported atrophy of the brainstem and cerebellum as well as the cerebral cortex. Clinical evaluation of magnetic resonance images (MRIs) in a small sample of young individuals (mean age 17.5 years) with WFS also revealed atrophy in the brainstem [14]. This finding was confirmed and extended by a recent, objectively quantified analysis of a larger and younger sample of people with WFS, which found reduced gray and white matter volumes and reduced white matter microstructural integrity within the brainstem and cerebellum as compared to age and gender comparable healthy and type 1 diabetic control groups [15]. As the cerebellum and brainstem have been commonly linked to gait and balance function [16-19], a clinical presentation of ataxic gait may be expected in WFS. However, until recently gait and balance abnormalities in WFS have only been vaguely and qualitatively described.

“Truncal ataxia causing unsteady gait and falls” [2] was reported but not quantified in 15 of 45 individuals with WFS in the late third and throughout the forth decade of life [2]. A more recent review of individual case histories found that clinically apparent gait abnormalities can appear in the second decade of life [3]. We have previously quantified balance performance in young individuals with WFS and found that balance impairment was common in these children and related to the severity of neurological soft signs rather than chronological age [20]. Based on these findings, we hypothesized that quantifiable gait impairments may also be detectable early in life in individuals with WFS compared to typically developing (TD) individuals.

Should gait deficits be present in individuals with WFS, understanding the underlying causes of the impairments would be helpful in informing treatment options. For example, gait impairments could be a direct result of deficits in balance [20]. Alternatively, gait impairments could result from deficits in visual acuity, which are common in individuals with WFS [4,21,22], and have been shown to impact typical gait development in other populations [23]. Finally, it is possible that the motor system is altered at an early age and leads to both balance and gait deficits [3,15]. Understanding this information will help characterize WFS neurological features, which may be the target of future pharmaceutical intervention. Furthermore, quantification of atypical gait patterns could be useful for developing physical therapy interventions to improve gait function in everyday life for people with WFS.

The objectives of this study were to: 1) compare spatiotemporal gait parameters of individuals with WFS to TD controls and 2) determine the relationship between visual acuity, balance, and gait impairment in individuals with WFS. We hypothesized that individuals with WFS would have decreased overground walking velocity, increased cadence, take shorter and wider steps, and spend a greater percentage of time in the double support phase as compared to TD controls. Further, we expected that both visual acuity and balance would be related to gait performance in individuals with WFS.

## Methods

### Participants

Thirteen individuals diagnosed with WFS (8 female, mean age 15.5 years, SD 6.3 yrs, min 6.4 yrs, max 25.8 yrs) and thirty TD young individuals (16 female, mean age 13.2 years, SD 6.2 yrs, min 5.6 yrs, max 28.5 yrs) participated. The Washington University Wolfram Syndrome Registry (http://wolframsyndrome.dom.wustl.edu/medical-research/Wolfram-Syndrome-Home.aspx) was used to recruit all individuals with WFS. Data were collected as part of an annual Wolfram Syndrome Research Clinic at the Washington University School of Medicine in St. Louis, Missouri. Balance and neuroimaging data from this clinic have been previously published [15,20]. All individuals with WFS had diabetes mellitus and optic atrophy before 18 years of age and genetic confirmation of a *WFS1* mutation. Exclusion criteria included being naïve to the diagnosis of WFS, complications of the disease which made travel difficult, or inability to participate in the majority of the research tests. Inclusion criteria for TD individuals included no known developmental delay or serious medical condition. Typically developing individuals were examined for neurological soft signs via the Physical and Neurological Examination for Subtle Signs (PANESS) [24], and were excluded if they presented more than two standard deviations below the mean for their age group. No TD individuals were excluded on this basis. Legal guardians provided informed written consent for all participants under age 18. All participants provided either informed written consent or assent prior to participation in accord with the procedures approved by the Human Research Protection Office of the Washington University School of Medicine.

Height, weight, and year in school were collected from all participants on the day of testing. Prior to beginning the gait trials, bilateral measures of leg length were collected by measuring from the greater trochanter to the lateral malleolus. For the WFS patients, best correctable visual acuity was measured for left and right monocular conditions as well as binocular vision using the distance Snellen visual acuity chart. Here we report only binocular visual data. One individual with WFS was not able to complete the visual testing due to diabetic complications. Blood glucose levels of the individuals with WFS were monitored by each individual’s care provider throughout the entirety of the Wolfram Syndrome Research Clinic. Readings were taken and recorded by study personnel at various points during the two day visit. Demographic data for each individual with WFS and summary data for the TD individuals are provided in Table 1.

**Table 1:** Clinical and demographic data for individuals with WFS and descriptive statistics for the typically developing (TD) group

**Gender**	**Age**	**School Year**	**Height (m)**	**Mass (kg)**	**Visual Acuity**	**Clinical Features (yrs)**
female	6.4	1	1.13	19.70	20/50	WFS(5), DM(4), DI(*), OA(5), HL(no)
male	8.3	3	1.24	24.90	20/20	WFS(3), DM(3), DI(7), OA(N/A), HL(no)
male	9.3	4	1.26	24.10	20/100	WFS(7), DM(7), DI(*), OA(6), HL(no)
female	11.9	7	1.64	43.70	20/70	WFS(9), DM(6), DI(7), OA(9), HL(no)
female	12.6	7	1.38	40.50	20/250	WFS(8), DM(6), DI(11), OA(7), HL(yes)
female	12.6	6	1.45	39.30	No data	WFS(8), DM(7), DI(11), OA(8), HL(no)
female	14.7	9	1.54	45.20	20/50	WFS(13), DM(3.9), DI(N/A), OA(13), HL(yes)
male	15.4	10	1.59	46.60	20/200	WFS(11), DM(10), DI(14), OA(11), HL(no)
female	17.1	12	1.58	87.90	20/25	WFS(16), DM(5), DI(N/A), OA(16), HL(no)
male	18.9	13	1.72	60.80	20/160	WFS(7), DM(5), DI(7), OA(7), HL(yes)
male	23.9	17	1.79	87.10	20/70	WFS(17), DM(7), DI(17), OA(7), HL(yes)
female	24.7	14	1.53	73.80	++	WFS(12), DM(2), DI(12), OA(5), HL(yes)
female	25.8	17	1.64	58.10	20/100	WFS(15), DM(13), DI(19), OA(13), HL(no)
WFS Mean (SD)	15.5 (6.3)	9.2 (5.2)	1.5 (0.20)	50.1 (22.5)		
TD Mean (SD)	13.4 (6.1)	7.8 (6.0)	1.5 (0.19)	46.4 (18.1)	n/a	N = 29 (16 females, 13 males)

WFS = Age of Wolfram syndrome diagnosis, DM = onset of diabetes mellitus, OA = onset of optic atrophy, DI = onset of diabetes insipidus, HL = presence of hearing loss.

* Not formally diagnosed but on DDAVP (desmopressin tablet) for enuresis.

++ individual able to detect hand motion at one foot.

Balance was assessed using the mini-Balance Evaluation Systems Test (mini-BESTest). This 14-item clinical battery is used to assess balance in four component areas (anticipatory transitions, postural response, sensory orientation and dynamic gait) and provides a single number summary of balance performance (maximum possible score = 32). These data have been previously reported in their entirety [22].

### Gait assessment

Kinematic features of gait were assessed on a 4.8 m GAITRite walkway system (CIR Systems, Havertown, Pennsylvania, USA), which has been called an “emerging clinical tool” for use with children [25] and has previously been used to quantify spatiotemporal gait parameters in typically developing children aged 1 to 10 years [26]. Each individual completed a minimum of five trials of the following five gait conditions: 1) forward walking at preferred speed (FP), 2) forward walking at a fast speed (FF), 3) forward walking while performing a dual task (FD), 4) forward tandem walking (FT), and 5) backward walking at preferred speed (BP). The order of the tasks was randomized, but all five trials of the given task were completed prior to beginning the next task. A start/finish line was placed one meter from each end of the mat to allow for capture of steady state walking without acceleration or deceleration. Participants were guarded during all trials to prevent falls and injury. Individuals with visual impairments were oriented to the testing space prior to beginning data collection. If the individual left the walkway prior to completion of the task, stumbled, or needed assistance from the spotter, the trial was discarded and immediately repeated. Each trial was initiated with a verbal cue and data recording began as soon as the individual contacted the GAITRite mat.

FP and BP walking were performed at each individual’s preferred speed with no cues relative to speed or completion time. For FF trials, each individual was instructed to walk as fast as possible without running. Dual task walking consisted of preferred-speed forward walking while listing as many items as possible in a given category. The categories consisted of ‘animals’, ‘things you see in the sky’, ‘games you play with a ball’, ‘colors,’ and ‘foods’. During these trials, the individual was accompanied closely by an investigator who recorded the number of correct, unique responses. The investigator did not initiate the trial and walked slightly behind the individual to avoid influencing the overground velocity of the participant. For tandem walking trials, individuals were instructed to walk with one foot in front of the other, as though they were on a tightrope or balance beam. Participants were instructed to walk with as much space between steps as was comfortable. Instructions directed the participants to not contact the heel of the front foot to the toe of the rear foot; however, once the trials began no corrective feedback was given. A blue, 3.8 cm wide, guide line was place down the center of the GAITRite mat to provide a visual target for the tandem task.

### Dependent variables

Five variables of interest were used to quantify unique components of gait in a clinically useful manner. Cadence, normalized velocity, normalized base of support, step extremity ratio and percentage of the gait cycle spent in double support time. These specific measures were selected as they are clearly defined, easily measured with appropriate technology, and can be addressed through rehabilitation intervention. Cadence, measured in steps per minute, was calculated by dividing the time needed to complete the trial by the number of steps taken. Velocity, measured in meters per second, was calculated by dividing the distance between initial and final footfall by the time to complete the trial. Due to the heterogeneity in height of the individuals, leg length measures were used to normalize velocity by dividing the velocity by the mean of the individual’s leg lengths. Base of support was defined as the perpendicular distance from the imaginary line connecting the center of the heel on one foot to the center of the heel on the opposite foot during two consecutive footfalls. Base of support was calculated by averaging the independently calculated values for the left and right sides. To generate a height normalized value, the average base of support value was divided by the individual’s height and multiplied by 100. The step extremity ratio is derived by dividing step length by leg length, and was computed by averaging both left and right side step extremity ratios. Finally, the percent of the gait cycle spent in double support is the duration of time the individual spent with both feet in contact with the floor divided by the total time needed to complete the trial and multiplied by 100.

**Figure 1:**
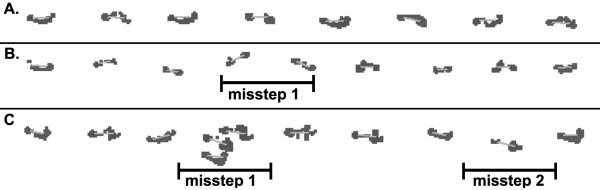
**Tandem gait trials were assessed by counting the number of times the individual had a footfall or series of footfalls off the linear path of progression. **(**A**) Trials in which all steps fell on the linear trajectory were scored as no missteps. Trials with one (**B**) or multiple (**C**) missteps were scored on the number of times the individual deviated from the linear trajectory and not on the number of individual steps that were off that trajectory.

Tandem walking trials could not be assessed using the GAITRite software due to a large number of parallel, overlapping, or backward steps. Therefore, tandem trials were scored based upon the number of times an individual experienced a loss of balance while traversing the walkway. A loss of balance was defined as any misstep or series of missteps which did not fall on the specified linear trajectory (Figure 1). Each trial was visually scored by viewing the entire length of the GAITRite walkway and manually counting the number of deviations from the subjectively assessed linear trajectory. A misstep was scored when a lateral deviation from the linear trajectory was noted.

### Data analysis

IBM© SPSS© Statistics Version 19 (IBM Corporation, Armonk, New York) was used for statistical analyses. For all conditions, trials with less than four consecutive, identifiable, full footfalls were excluded from analysis. 36 total trials were discarded (9 trials from 6 WFS subjects, 27 trials from 15 TD subjects). Data from one TD individual (age 5.6) could not be used due to poor footfall data on more than half of the recorded trials. These data have been removed from all portions of the analysis including the demographic description of participants. Additionally, dual task walking data from a second TD child (age 11.15) were removed as she could not complete the task without coming to a full stop multiple times during the trial. Data were averaged across valid trials for each condition. All individuals had a minimum of three valid trials for each condition.

The Shapiro-Wilk test was used to test the data for normal distribution. Demographic variables were not normally distributed (Shapiro Wilk p<.05), so groups were compared on these variables with non-parametric statistics (independent sample Mann Whitney U).

All spatiotemporal gait measures were normally distributed, thus parametric statistics were applied. A group (TD and WFS) by task (FP, FF, FD and BP) mixed model analysis of variance (ANOVA) was used to test for differences both within and between groups in each spatiotemporal gait measure separately (cadence, normalized velocity, step extremity ratio, normalized base of support, the percent of the gait cycle spent in double support). A Chi-square test was used to compare the percentage of individuals in each group who had at least one misstep during tandem walking. An a priori level of α<0.05 was set for determining statistical significance.

Bivariate correlations were used to assess the relationship between age and spatiotemporal gait variables. P-values and Spearman’s correlation coefficient (r_s_) are reported for all correlations. An a priori level of α<0.05 was set for determining statistical significance. Within the WFS group, bivariate correlations were used to assess the relationship between visual acuity, balance [20] and spatiotemporal gait measures.

## Results

Demographic data for both groups are shown in Table 1. WFS and age-matched controls did not differ by age (Z = −1.08, p = 0.28), height (Z = −0.59, p = 0.56), weight (Z = 0.37, p = 0.71), or year in school (Z = 1.06, p = 0.29). Additionally, the average number of verbal responses generated during dual task walking trials for TD and WFS did not differ significantly (F = 0.408, p = 0.53) between groups. Mean blood glucose levels taken during the study for the individuals with WFS averaged 222.5 mg/dL (SD = 69.1 mg/dL, max = 312.5 mg/dL, min = 94 mg/dL). No patients experienced any acute neurological symptoms related to hypoglycemia or hyperglycemia during the study.

### Between group spatiotemporal gait data

**Figure 2:**
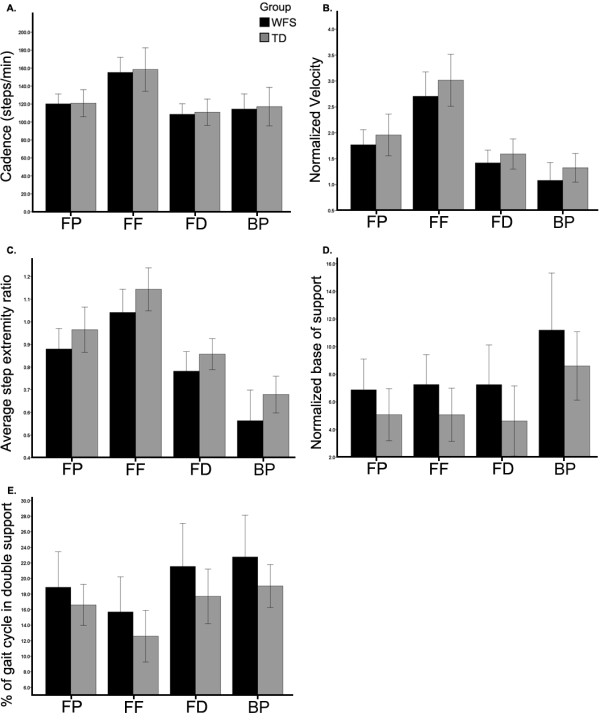
**Cadence (A), normalized velocity (B), average normalized base of support (C), average step extremity ratio (D) and percentage of gait cycle spent in double support (E) of individuals with WFS (black bars) and TD individuals (gray bars) during forward preferred, forward fast, dual task and backward walking tasks. **Cadence values did not differ between groups. Group level differences were present for all other spatiotemporal measures. Values are mean +/− SD.

A main effect of group was found for all gait measures except cadence (F = 0.25, p = 0.62). Individuals with WFS walked consistently slower (normalized velocity, F = 5.2, p ≤ 0.03), took relatively shorter steps (step extremity ratio, F = 14.7, p ≤ 0.001) with a wider normalized base of support (F = 14.2, p = 0.001) and had a greater amount of the gait cycle spent in double support (F = 10.2, p = 0.003) than TD individuals (Figure 2).

### Between task spatiotemporal gait data

A significant main effect of task was present for all measures (p ≤ 0.001). During FF walking trials, individuals averaged the highest cadence (cadence = 157.2, SD = 22.4), fastest velocity (unitless normalized velocity = 2.9, SD = 0.05), largest step extremity ratio (step extremity ratio = 1.09, SD = 0.02) and the smallest percent of the gait cycle spent in double support (percent double support = 14.3, SD = 0.6). The largest normalized base of support occurred during backward walking (unitless normalized base of support = 9.9, SD = 0.5), while the smallest normalized base of support occurred during dual task forward walking (unitless normalized base of support = 5.9, SD = 0.5). Dual task forward walking also had the fewest steps per minute (cadence = 110.2, SD = 13.8). Backward walking trials had the slowest (unitless nVel = 1.3, SD = 0.31), shortest steps (step extremity ratio = 0.62, SD = 0.02), widest base of support (unitless normalized base of support = 9.9, SD = 0.5) and longest percent of the gait cycle spent in double support (percent double support = 20.9, SD = 0.6). No group by task interactions were present (Table 2).

**Table 2:** Main effects of group and task and two-way interactions

		**Cadence (steps/min)**	**p-value**	**Normed Velocity**	**p-value**	**Step Extremity Ratio**	**p-value**	**Normed Base of Support**	**p-value**	**% Double Support**	**p-value**
group	WFS	124.4 ± 4.4	= 0.620	1.7 ± 0.08	* = 0.028	0.82 ± 0.02	** < 0.001	8.2 ± 0.5	** = 0.001	20.0 ± 0.9	** = 0.003
	TD	127.1 ± 2.9		2.0 ± 0.06		0.91 ± 0.01		5.8 ± 0.4		16.5 ± 0.6	
task	FP	121.3 ± 13.7	** ≤ 0.001	1.9 ± 0.38	** ≤ 0.001	0.92 ± 0.02	** ≤ 0.001	6.1 ± 0.3	** ≤ 0.001	17.9 ± 0.6	** ≤ 0.001
	FF	157.2 ± 22.4		2.9 ± 0.52		1.09 ± 0.02		6.2 ± 0.3		14.3 ± 0.6	
	FD	110.2 ± 13.8		1.5 ± 0.29		0.82 ± 0.01		5.9 ± 0.5		19.9 ± 0.7	
	BP	116.3 ± 20.0		1.3 ± 0.31		0.62 ± 0.02		9.9 ± 0.5		20.9 ± 0.6	
		group x task									
WFS	FP	120.5 ± 11.4	= 0.936	1.8 ± 0.30	= 0.604	0.89 ± 0.03	= 0.603	7.2 ± 0.6	= 0.948	19.2 ± 1.0	= 0.462
	FF	154.0 ± 16.9		2.7 ± 0.48		1.04 ± 0.03		7.3 ± 0.6		16.0 ± 1.1	
	FD	108.8 ± 12.2		1.4 ± 0.26		0.78 ± 0.02		7.2 ± 0.8		22.1 ± 1.2	
	BP	114.4 ± 16.8		1.1 ± 0.34		0.56 ± 0.03		11.2 ± 0.9		22.8 ± 1.1	
TD	FP	121.7 ± 14.7		2.0 ± 0.41		0.96 ± 0.02		5.1 ± 0.4		16.6 ± 0.6	
	FF	158.6 ± 24.6		3.0 ± 0.51		1.14 ± 0.02		5.0 ± 0.4		12.7 ± 0.7	
	FD	110.9 ± 14.6		1.6 ± 0.29		0.86 ± 0.01		4.6 ± 0.5		17.7 ± 0.8	
	BP	117.1 ± 21.5		1.3 ± 0.28		0.68 ± 0.02		8.6 ± 0.6		19.0 ± 0.7	

WFS = individuals with Wolfram syndrome; TD = typically developing individuals; FP = forward preferred; FF = forward fast; FD = forward dual task; and BP = backward preferred.

Values are means ± SD.

* denotes significant correlation at the 0.05 level.

** denotes significant correlation at the 0.01 level.

### Tandem walking

The groups differed in the number of missteps during tandem walking (*F* = 28.4, p ≤ 0.001). On average, healthy individuals lost their balance less than one time per ten tandem walking trials (mean misstep per trial = 0.03, SD = 0.08), while individuals with WFS averaged over seven deviations per ten trials (mean misstep per trial = 0.72, SD = 0.71). Individuals with WFS were significantly more likely to experience a misstep than TD individuals (*χ*^2^ = 15.3, df =1, p≤ 0.001). Nine of 13 (69 %) of the individuals with WFS but only three of 29 healthy individuals (10 %) experienced one or more losses of balance during the tandem walking trials.

### Age

No significant correlations were present between age and any of the normalized spatiotemporal gait measures (i.e., normalized velocity, step extremity ratio and normalized base of support) for individuals with WFS (Table 3). During FP walking trails, age was correlated to normalized velocity (r_s_= −0.57, p ≤ 0.001) and step extremity ratio (r_s_= −0.47, p = 0.010) for TD individuals. Additionally, in TD individuals age was correlated to normalized velocity (r_s_= −0.70, p ≤ 0.001) forward fast walking trials. All other correlations of age and normalized spatiotemporal gait measures failed to achieve significance (Table 3).

**Table 3:** Significance (top) and correlation coefficients (bottom) between age (years) and gait measures for each task

				**Cadence (steps/min)**	**Normalized Velocity**	**Step Extremity Ratio**	**Normalized Base of Support**	**% Double Support**
**WFS**	FP	Age	Sig. (2-tailed)	0.642	0.388	0.279	0.859	0.045 *
			r_s_	0.143	0.262	0.325	−0.055	0.564
	FF	Age	Sig. (2-tailed)	0.150	0.405	0.873	0.929	0.128
			r_s_	−0.423	−0.253	−0.049	−0.027	0.445
	FD	Age	Sig. (2-tailed)	0.845	0.529	0.674	0.915	0.006 **
			r_s_	−0.060	−0.192	−0.129	0.033	0.720
	BP	Age	Sig. (2-tailed)	0.854	0.318	0.199	0.366	0.198
			r_s_	0.060	0.315	0.399	0.287	0.399
**TD**	FP	Age	Sig. (2-tailed)	0.000 **	0.001 **	0.010 *	0.056	0.000 **
			r_s_	−0.648	−0.574	−0.470	−0.359	0.703
	FF	Age	Sig. (2-tailed)	0.000 **	0.000 **	0.359	0.337	0.003 **
			r_s_	−0.695	−0.703	−0.177	−0.185	0.539
	FD	Age	Sig. (2-tailed)	0.016 *	0.062	0.799	0.814	0.000 **
			r_s_	−0.451	−0.357	0.050	0.047	0.706
	BP	Age	Sig. (2-tailed)	0.002 **	0.119	0.282	0.451	0.011 *
			r_s_	−0.549	−0.301	0.211	0.148	0.475

* denotes significant correlation at the 0.05 level.

** denotes significant correlation at the 0.01 level.

**Figure 3:**
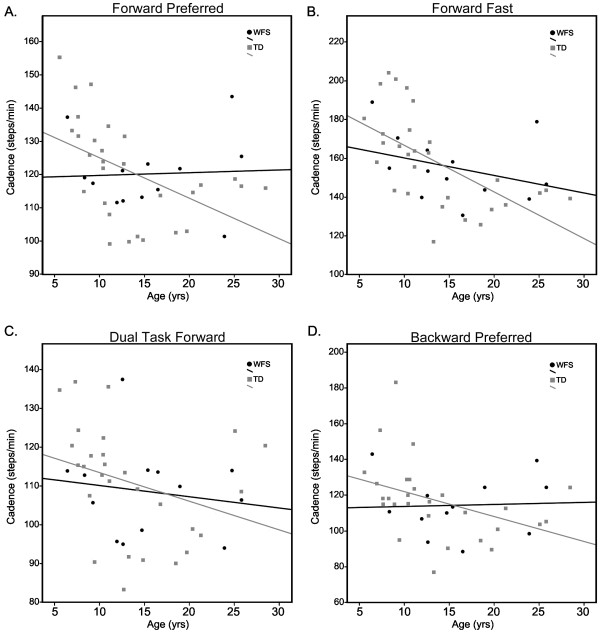
**Cadence versus age for individuals with WFS (black) and TD individuals (gray) during (A) forward preferred, (B) forward fast, (C) dual task and (C) backward walking tasks. **Cadence was significantly correlated with age in all four task conditions for the TD individuals. Cadence was not significantly correlated with age for individuals with WFS in any condition.

In the TD group, cadence was significantly correlated to age during all gait tasks. Cadence was not correlated with age for individuals with WFS during any of the tested tasks (Figure 3). Age and percent of the gait cycle spent in double support were significantly correlated in TD individuals across all gait tasks and in WFS during FP and FD tasks (Table 3).

### Vision, balance and gait

Visual acuity scores for the WFS group are reported in Table 1. Two individuals were not able to fully complete the vision test. One missed the second day of testing due to diabetic complications and the other could not successfully perform the vision assessment as her vision was too impaired. Data presented here are from the remaining eleven individuals.

Within the WFS group, individual visual acuity scores were not correlated with cadence, normalized velocity, normalized base of support, step extremity ratio or the percent of the gait cycle spent in double support during any of the gait tasks (Table 4). Visual acuity was also not correlated with the number of missteps during tandem walking (r_s_=0.44, p = 0.18).

**Table 4:** Significance (top) and correlation coefficients (bottom) between visual and gait measures for each gait task

			**Cadence (steps/min)**	**Normalized Velocity**	**Step Extremity Ratio**	**Normalized Base of Support**	**% Double Support**
FP	Visual Acuity	Sig. (2-tailed)	0.34	0.85	0.53	0.58	0.43
		r_s_	0.32	−0.06	−0.21	−0.19	−0.27
FF	Visual Acuity	Sig. (2-tailed)	0.46	0.98	0.60	0.44	0.36
		r_s_	0.25	0.01	−0.18	−0.26	−0.31
FD	Visual Acuity	Sig. (2-tailed)	0.45	0.98	0.21	0.29	0.72
		r_s_	0.26	−0.01	−0.41	−0.35	0.12
BP	Visual Acuity	Sig. (2-tailed)	0.26	0.35	0.66	0.24	0.85
		r_s_	0.40	0.33	0.16	−0.41	−0.07

**Figure 4:**
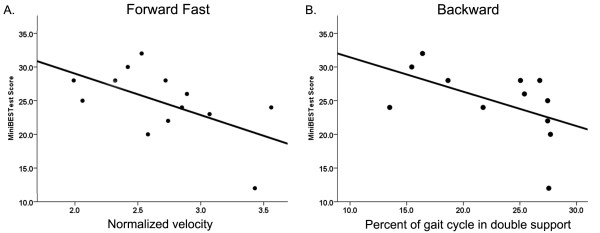
**Significant correlations between mini-BESTest and (A) normalized velocity during forward fast gait and (B) percentage of the gait cycle spent in double support for the WFS group. **Higher mini-BESTest scores correspond to better balance.

Balance data from the mini-BESTest for all thirteen individuals with WFS were previously reported [20]. Visual acuity was not correlated with mini-BESTest scores (r_s _= −0.57, p = 0.19). Individual balance scores were negatively correlated with normalized velocity in the FF task (r_s_ = −0.59, p = 0.03), indicating that individuals with worse balance walked with a faster normalized velocity (Figure 4). The percent of the gait cycle spent in double support during backward walking was also negatively correlated with mini-BESTest scores (r_s _= −0.64, p = 0.03), indicating that individuals with worse balance spent a greater percentage of the gait cycle in double support during backward walking (Figure 4). Mini-BESTest scores did not correlate with any other spatiotemporal measure (cadence, normalized base of support or step extremity ratio) during any of the gait tasks (Table 5). Mini-BESTest scores were not correlated with number of missteps during tandem walking (r_s_ = −0.48, p = 0.10).

**Table 5:** Significance (top) and correlation coefficients (bottom) between mini-BESTest scores and gait measures

			**Cadence (steps/min)**	**Normalized Velocity**	**Step Extremity Ratio**	**Normalized Base of Support**	**% Double Support**
FP	mini-BESTest	Sig. (2-tailed)	0.94	0.55	0.74	0.59	0.55
		r_s_	−0.03	0.18	0.10	0.17	−0.18
FF	mini-BESTest	Sig. (2-tailed)	0.13	0.03 *	0.12	0.59	0.87
		r_s_	−0.45	−0.59	−0.46	0.16	0.05
FD	mini-BESTest	Sig. (2-tailed)	0.83	0.78	0.86	0.49	0.51
		r_s_	−0.07	−0.09	0.05	0.21	0.20
BP	mini-BESTest	Sig. (2-tailed)	0.86	0.42	0.38	0.21	0.03 *
		r_s_	−0.06	0.26	0.28	−0.39	−0.64

* denotes significant correlation at the 0.05 level.

** denotes significant correlation at the 0.01 level.

## Discussion

The goal of this study was to quantify spatiotemporal gait function in young individuals with WFS and compare these individuals to a group of TD individuals. Although ataxic gait has been noted in clinical reports of WFS [2,3], to our knowledge, no study aside from our own prior work focused on balance deficits [20] has attempted to quantify motor deficits in the WFS population. As hypothesized, individuals with WFS walk significantly slower, take shorter and wider steps, spend more time in double support and have more difficulty performing tandem walking compared to typically developing individuals. Deficits in the WFS groups were present across all tested gait tasks. These differences do not appear to be directly associated with age, visual deficits or impaired balance in WFS patients. These findings indicate that gait is measurably and independently impaired in young individuals with WFS.

As compared to the TD group, the WFS group showed significant deficits in normalized velocity, normalized base of support, step extremity ratio and the percent of the gait cycle spent in double support across all gait tasks. The spatiotemporal gait abnormalities in WFS may indicate the use of compensatory strategies for decreased balance. Supporting this idea, individuals with WFS were more likely to experience a loss of balance resulting in a lateral deviation during tandem walking. Similarly, voluntary changes in step width and length have been shown to alter the stability of trunk motion [27]. In an examination of turning strategies of individuals with cerebellar ataxia, Mari et al. [28] showed that during a demanding gait task, such as turning, ataxic patients displayed wider and shorter steps. The authors theorized that the observed gait difference between healthy and ataxic individuals was a compensatory strategy used to reduce instability. Slower walking velocity has also been previously been linked to increased stability [29] indicating that individuals will compensate for a loss of stability via a decrease in gait velocity. Dingwell et al. [30] noted that individuals with diabetic peripheral neuropathy demonstrate slower walking speeds and increased dynamic stability as compared to healthy, age-matched individuals. They concluded that decreased walking speed is a compensatory strategy used by the patient group to maintain stability during walking. As such, we hypothesized that the observed deficits in spatiotemporal gait measures would be related to previously reported balance deficits in individuals with WFS.

We have previously reported a deficit in balance in individuals with WFS [20], particularly during tasks completely reliant on balance and on which compensatory strategies cannot be employed, such as standing on one foot or standing on an unstable surface. In contrast, during the dynamic gait subsection of the mini-BESTest which relies on gait-related balance tasks such as changing velocity and turning the head while walking, no deficits were detected in WFS. Perhaps this is because they were able to employ compensatory strategies during this group of tasks that could not be used during more static balance tasks, such as a wider stance. However, if the observed differences in gait between WFS and TD individuals are the result of strategies used to compensate for balance deficits, a clear association between balance and spatiotemporal gait measures would be expected. However, there appears to be no consistent correlation between spatiotemporal gait measures and previously reported balance scores, suggesting that other factors should be explored.

Only two spatiotemporal gait features, normalized fast forward gait velocity and double support during backward walking, were significantly correlated with balance. The significant negative correlation between mini-BESTest score and normalized velocity was unexpected. This indicates that individuals with WFS with lower mini-BESTest scores (worse balance) performed FF walking trials at higher velocity than individuals with WFS and higher mini-BESTest scores (better balance). This result is strongly driven by the performance of a single individual who scored poorly on the mini-BESTest but was able to walk at a fast velocity. We are only able to speculate at this time, but this type of high velocity gait with low overall balance may lead to an increased number of falls. Future studies should include a falls assessment to address this question and may be helpful in identifying the needs of the patient group.

In addition to balance, vision has been repeatedly linked to locomotor performance [31,32]. Specifically, in young people, visual impairment correlates with spatiotemporal gait deficits [23,33]. In WFS, visual deficits are well described (for review see [34]) thus we expected gait impairments in individuals with WFS to correlate with measures of visual impairment. However, we did not find any correlation between visual acuity and any spatiotemporal measures of gait function, suggesting that vision may not be a key factor in the gait differences noted in WFS compared to TD individuals. Here again, as with correlations to measures of balance, the small sample size should be noted.

Previous reports indicate that children may exhibit mature gait patterns as early as four years of age [35], though others argue that gait continues to follow a developmental trend and does not reach adult-like patterns until after the seventh year and perhaps even beyond the tenth year of age [36]. As our sample spans from five to 26 years, age-related changes in spatiotemporal gait measures cannot be overlooked. Three of the selected measures (velocity, base of support and step length) were normalized to account for anatomically related developmental changes such as increased leg length and height. Within the WFS group, no significant correlations were present between age and these normalized measures. In the TD group, only three of the twelve normalized variables examined were significantly correlated with age. However cadence, a non-normalized variable, was significantly negatively correlated with age across all gait tasks in the TD group but not in the WFS group. This matches our previous report of significant correlations between balance and age in the TD group but not in the WFS group [20]. This lack of a clear relationship between age and cadence within the WFS population may indicate that individuals with WFS are neurologically affected early in their lifespan and thus do not conform to normal gait developmental patterns. This notion is further supported by the early presence of reduced gray and white matter volumes and reduced white matter microstructural integrity within the brainstem and cerebellum as compared to age and gender comparable healthy and type 1 diabetic control groups [15]. However, caution is warranted as the sample of individuals with WFS in the present study is relatively small.

Based upon the current findings, gait impairments cannot be accounted for by the particular sensory or motor deficits we evaluated. Additionally, age does not consistently relate to spatiotemporal gait measures in WFS. The overall model of neurological symptom presentation and the corresponding treatment approach to WFS is complicated by the observed deficits in multiple sensory and motor systems as well as the unique phenotypic presentation of these symptoms for each patient diagnosed with WFS. To date, the sensory and motor characteristics of individuals with WFS have been primarily qualitatively described. Clinically noted deficits have been reported but are rarely quantified. Longitudinal data quantitatively tracking neurological symptoms in a larger sample across the lifespan will be necessary to fully address this question. However, the presentation of early gait and balance deficits, along with the presence of early abnormalities of the brainstem and cerebellum indicates that neurological symptom presentation occurs in early childhood, and could be addressed clinically at an early age.

### Limitations

As the present investigation includes only 13 individuals with WFS and these individuals span the range of six to twenty-six years of age, a complex multimodal assessment of symptom presentation and compensatory strategy is beyond the scope of this study. Longitudinal data tracking symptom progression and subsequent gait changes would allow for an ideal and novel examination of disease progression and could inform targeted drug and clinical interventions.

## Conclusions

Wolfram Syndrome is rare and debilitating with neurological motor symptom onset occurring far earlier than previously reported. We have quantified deficits in normalized velocity, stride length, normalized base of support, and the percent of the gait cycle spent in double support in a group of young individuals with WFS. These data provide evidence of the early emergence of gait abnormalities in WFS.

## Competing interests

The authors declare that they have no competing interests.

## Authors’ contributions

KP participated in the design of the study, collected the data, performed the statistical analysis and drafted the manuscript. RD participated in the design of the study, collected the data and helped to draft the manuscript. JH collected data and helped to draft the manuscript. BM was the medical director of the larger annual Wolfram Syndrome Research Clinic, collected data and helped to draft the manuscript. TH conceived of the study, participated in the design of the study, oversaw execution of the larger annual Wolfram Syndrome Research Clinic and helped to draft the manuscript. GE participated in the design of the study, collected the data and helped to draft the manuscript. All authors read and approved the final manuscript.

## Authors’ information

Washington University Wolfram Study Group Members: In addition to the authors: P. Austin, M.D., A. Bondurant, B.A., T. Hullar, M.D., R. Karzon, Ph.D., J. Lapp, B.A., J. Leey M.D., H. M. Lugar M.A., L. Manwaring, M.S., C. Nguyen, B.S., A.R. Paciorkowski M.D., J. Rutlin, J. Shimony, F. Urano, M.D., Ph.D., A. Viehoever, M.D., J. Wasson B.S., and N. H. White M.D., CDE.
